# The ML-EM Algorithm is Not Optimal for Poisson Noise

**DOI:** 10.1109/NSSMIC.2015.7582178

**Published:** 2015

**Authors:** Gengsheng L. Zeng

**Affiliations:** The author is with the Department of Engineering, Weber State University, Ogden, UT 84408 USA, and also with the Department of Radiology, University of Utah, Salt Lake City, UT 84108 USA

**Keywords:** Computed tomography, expectation maximization (EM), iterative reconstruction, maximum likelihood (ML), noise weighted image reconstruction, Poisson noise, positron emission tomography (PET), single photon emission computed tomography (SPECT)

## Abstract

The ML-EM (maximum likelihood expectation maximization) algorithm is the most popular image reconstruction method when the measurement noise is Poisson distributed. This short paper considers the problem that for a given noisy projection data set, whether the ML-EM algorithm is able to provide an approximate solution that is close to the true solution. It is well-known that the ML-EM algorithm at early iterations converges towards the true solution and then in later iterations diverges away from the true solution. Therefore a potential good approximate solution can only be obtained by early termination. This short paper argues that the ML-EM algorithm is not optimal in providing such an approximate solution. In order to show that the ML-EM algorithm is not optimal, it is only necessary to provide a different algorithm that performs better. An alternative algorithm is suggested in this paper and this alternative algorithm is able to outperform the ML-EM algorithm.

## I. Introduction

The ML-EM (maximum likelihood expectation maximization) algorithm became popular in the field of medical image reconstruction in the 1980s [1][2]. This algorithm considers the Poisson noise model and finds wide applications in PET (positron emission tomography) and SPECT (single photon emission computed tomography). Besides Poisson noise modeling, it also guarantees that the resultant image is non-negative and the total photon count of the forward projections of the resultant image is the same as the total photon count of the measured projections.

It is known that as the iteration number of the ML-EM algorithm increases, the likelihood objective function monotonically increases. The ML-EM algorithm will converge to a maximum likelihood solution. However, the convergence of the algorithm does not imply that the converged image is close to the true image. In fact, the ML-EM algorithm first converges towards the true solution, and then diverges away from it. The final maximum likelihood solution is too noisy to be useful. One approach is to apply a post lowpass filter on the finally converged noisy image, but one has to determine an *ad hoc* lowpass filter. Another approach is to stop the algorithm early. If the algorithm is terminated at the right time, a good approximate solution is close to the true solution.

This short paper argues that the ML-EM algorithm is not optimal in providing such an approximate solution. In order to show that the ML-EM algorithm is not optimal, it is only necessary to provide a different algorithm that performs better. An alternative algorithm is suggested in this paper and this alternative algorithm is able to outperform the ML-EM algorithm.

## II. Methods

Let $x_{i}^{\left(x\right)}$ be the *i*th image pixel at the *n*th iteration, *p_j_* be the *j*th line-integral (ray-sum) measurement value, *a_ij_* be the contribution of the *i*th image pixel to the *j*th measurement, and $q_{j}^{\left(n\right)}=\sum_{i}a_{ij}x_{i}^{\left(n\right)}$ be the forward projection of the image at the *n*th iteration, then the ML-EM algorithm can be expressed as [1][2]

(1) $$x_{i}^{\left(n+1\right)}=x_{i}^{\left(n\right)}\frac{\sum_{j}a_{ij}\frac{p_{j}}{q_{j}^{\left(n\right)}}}{\sum_{j}a_{ij}},$$

where the summation over the index *j* is the backprojection. This algorithm can be extended into a more general algorithm by introducing a new parameter *α*:

(2) $$x_{i}^{\left(n+1\right)}=x_{i}^{\left(n\right)}\frac{\sum_{j}a_{ij}\frac{p_{j}}{{\left[q_{j}^{\left(n\right)}\right]}^{\alpha }}}{\sum_{j}a_{ij}\frac{q_{j}^{\left(n\right)}}{{\left[q_{j}^{\left(n\right)}\right]}^{\alpha }}}.$$

When *α* = 1, Algorithms (2) and (1) are identical. It is obvious that Algorithm (2) produces non-negative images. The motivation of introducing the parameter *α* is to change the noise weighting factor for each measurement *p_j_*. This can be seen more clearly by expressing (2) in the additive form [3]

(3) $$x_{i}^{\left(n+1\right)}=x_{i}^{\left(n\right)}\frac{\sum_{j}a_{ij}\frac{p_{j}}{{\left[q_{j}^{\left(n\right)}\right]}^{\alpha }}}{\sum_{j}a_{ij}\frac{q_{j}^{\left(n\right)}}{{\left[q_{j}^{\left(n\right)}\right]}^{\alpha }}}=x_{i}^{\left(n\right)}\frac{\sum_{j}a_{ij}\frac{p_{j}-q_{j}^{\left(n\right)}+q_{j}^{\left(n\right)}}{{\left[q_{j}^{\left(n\right)}\right]}^{\alpha }}}{\sum_{j}a_{ij}\frac{q_{j}^{\left(n\right)}}{{\left[q_{j}^{\left(n\right)}\right]}^{\alpha }}}=x_{i}^{\left(n\right)}\frac{\sum_{j}a_{ij}\frac{q_{j}^{\left(n\right)}}{{\left[q_{j}^{\left(n\right)}\right]}^{\alpha }}}{\sum_{j}a_{ij}\frac{q_{j}^{\left(n\right)}}{{\left[q_{j}^{\left(n\right)}\right]}^{\alpha }}}+x_{i}^{\left(n\right)}\frac{\sum_{j}a_{ij}\frac{p_{j}-q_{j}^{\left(n\right)}}{{\left[q_{j}^{\left(n\right)}\right]}^{\alpha }}}{\sum_{j}a_{ij}\frac{q_{j}^{\left(n\right)}}{{\left[q_{j}^{\left(n\right)}\right]}^{\alpha }}}=x_{i}^{\left(n\right)}+\frac{x_{i}^{\left(n\right)}}{\sum_{j}a_{ij}\frac{q_{j}^{\left(n\right)}}{{\left[q_{j}^{\left(n\right)}\right]}^{\alpha }}}\sum_{j}a_{ij}\frac{1}{{\left[q_{j}^{\left(n\right)}\right]}^{\alpha }}[p_{j}-q_{j}^{\left(n\right)}].$$

The last equation in (3) is in the form of an additive gradient decent algorithm, which is used to minimize an objective function:

(4) $$F=\sum_{j}W_{j}{\left(\sum_{i}a_{ij}x_{i}-p_{j}\right)}^{2}.$$

In (3), the step-size is

(5) $$\text{Step size}=\frac{x_{j}^{\left(n\right)}}{\sum_{j}a_{ij}q_{j}^{\left(n\right)}/{\left[q_{j}^{\left(n\right)}\right]}^{\alpha }}$$

and the noise weighting factor is

(6) $$W_{j}=\text{Noise weighting factor}=\frac{1}{{\left[q_{j}^{\left(n\right)}\right]}^{\alpha }}.$$

Here $q_{j}^{\left(n\right)}$ is the forward projection of the image and is the approximation of the mean value of the corresponding projection measurement. The mean value is the same as the variance for the Poisson distribution. Therefore, by changing parameter *α*, the noise model is changed, and the step-size is also affected. The “correct” model for Poisson noise is *α* = 1. We need to point out that algorithm (3) is not exactly the traditional gradient descent algorithm, because the weighting factor *W_j_* is allowed to vary from iteration to iteration as indicated by (6).

The ML-EM algorithm guarantees to converge to the noisy maximum likelihood solution, but it is not guaranteed to reach the best possible solution (i.e., closest to the true solution for the given data set) if the algorithm is terminated early at a certain iteration number.

The computer simulations in this paper used analytically generated projections and the object was not pixelized. The phantom was a large uniform ellipse containing 5 small hot uniform discs and 1 small cold uniform disc. The activity ratios of ellipse: hot: cold are 1: 2: 0.5. The Poisson noise was incorporated in the projections with 5 different noise levels with scaling factors of 0.1, 1, 10, 100, and 1000; the corresponding total photon counts in these 5 noise levels of measurements were approximately 10^6^, 10^7^, 10^8^, 10^9^, and 10^10^, respectively. The parallel beam imaging geometry was assumed, with 120 views over 360 and 128 detector bins at each view. The images were reconstructed in a 128 × 128 array.

A series of computer simulations is conducted in this paper to investigate how to reach the best solution by varying the parameter *α* and the iteration number *n*. By “best” it is meant that the mean-square-error (MSE) between the true image and the reconstructed image reaches the minimum among sampled parameter *α* and *n*. The mean-square-error is defined as

(7) $$\text{MSE}=\frac{1}{N}\sum_{i\in \text{support}}{\left(x_{i}-{\text{true}}_{i}\right)}^{2},$$

where *true_i_* is the *i*th pixel of the true image and *N* can be any fixed number. The product of the number of image pixels and the phantom scaling factor was used for *N* in this paper. In addition to MSE, a rectangular uniform region in the phantom is selected and the image standard deviation value is calculated in this rectangular region. The standard deviation value is normalized by the phantom scaling factor. The standard deviation value ignores the image bias, while MSE does not.

## III. Results

The implementation of the Algorithm (2) is almost the same as that of the conventional ML-EM algorithm (1), except that each iteration requires two backprojections instead of one. Since the true image is available, the algorithm is stopped when the MSE starts to increase for a given parameter *α*. The parameter *α* was sampled from 0.1 to 1.9 with a sampling interval of 0.1. The results for the 5 noise levels are listed in Tables I through V, respectively. For each noise level, results of 5 noise realizations are shown. The images shown are from one noise realization.

Each image in this paper is displayed individually from its minimum to its maximum using a linear gray scale. The images in each noise level look similar, but their numerical MSE values differ. In each table, central horizontal line profiles are provided to compare the image resolution. In order to visualize the important resolution information from the line profiles, the random noise from the projection data was removed while the parameter and the number of iterations were kept the same.

**Fig. 1:**
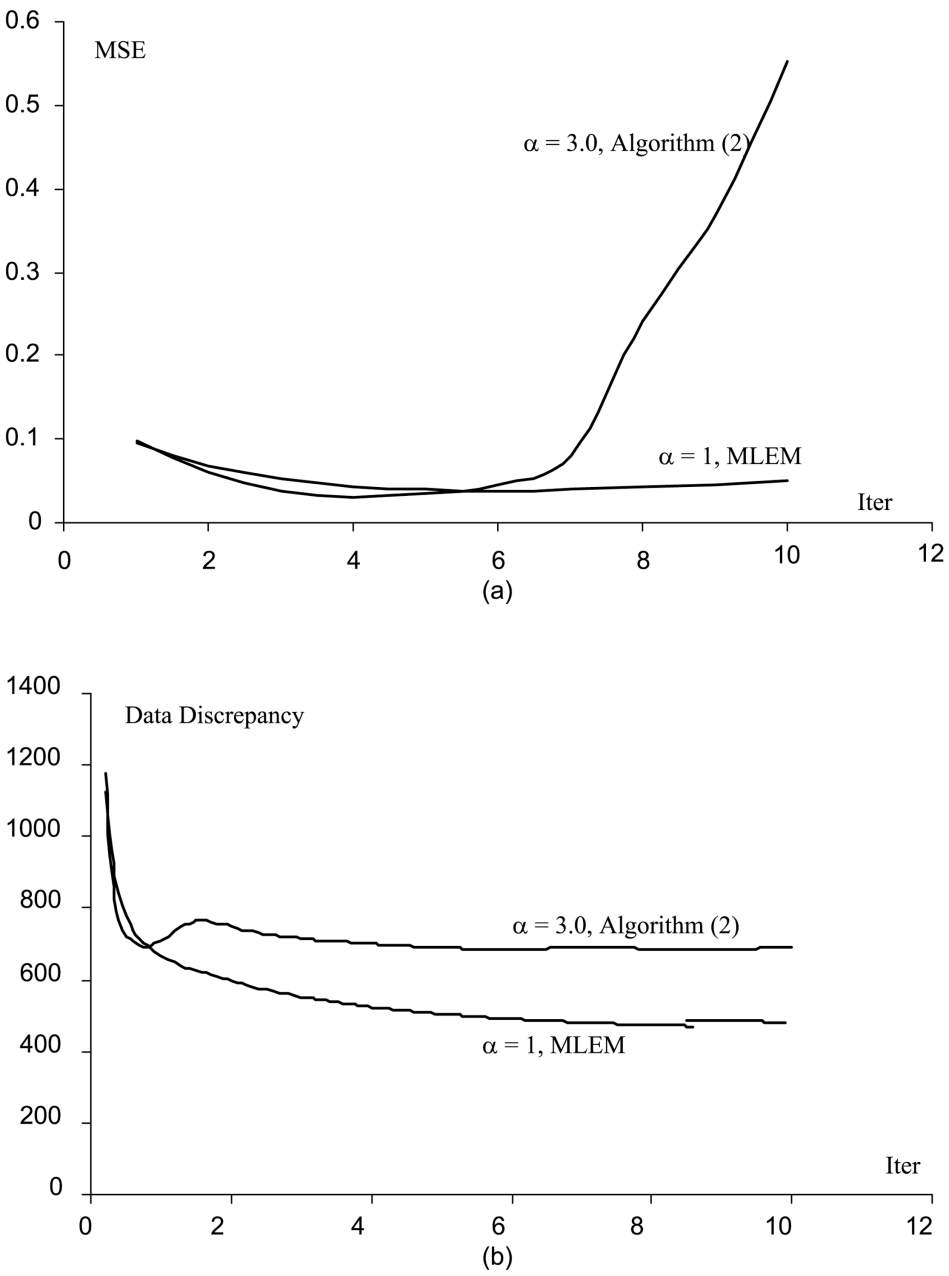
(A) MSE vs. iteration number curves for the MLEM and the Algorithm (2), when the phantom scaling factor is 0.1 (B) Data fidelity error vs. iteration number curves for the MLEM and the Algorithm (2), when the phantom scaling factor is 1.

Fig. 1(A) through 5(A) show the well-known phenomenon that the MSE initially decreases as the iteration number increases, while later the MSE increases. Fig. 1(B) through 5(B) show the trend that the projection data discrepancy error decreases as the iteration number increases. The data discrepancy is calculated by the squared error between the forward projection of the image and the measured data.

## IV. Discussion and Conclusions

When the projection measurement noise is Poisson distributed, the ML-EM algorithm is able to provide the maximum likelihood solution, which is noisy and has a large MSE from the true image. If the ML-EM algorithm stops early, it can provide solutions with a smaller mean-square-error (MSE) from the true image. This paper investigates whether the ML-EM algorithm is able to provide the best approximate solution (among all algorithms) when the algorithm can stop early. The result is negative. The ML-EM algorithm is not the optimal choice in this situation. The extended Algorithm (2) can outperform the conventional ML-EM algorithm (1). The unique feature of the extended Algorithm (2) is a new parameter *α*, which controls the noise model. When *α* = 1, the noise model is Poisson and the extended algorithm is identical to the conventional ML-EM algorithm (1).

A trend is observed from Tables 1 ∼ 5 that when data total count is lower, the optimal number of iterations is smaller and the optimal parameter *α* is larger (can be greater than 1). When data total count is higher, the optimal number of iterations is larger and the optimal parameter *α* is smaller (can be smaller than 1). This observation can be used as a general guidance that user can follow to choose the right *α* for clinical cases based on their study count level.

Numerical analysis is a branch of computational mathematics. It focuses on whether an algorithm converges, how fast it converges, and how stable the solutions are. Little attention is paid to the intermediate solutions by stopping an algorithm early. However, in ML-EM reconstruction, one of those intermediate solutions (not the final converged solution) has a small MSE with respect to the true image. However, an intermediate solution from a different algorithm (2) with a slightly different (and “incorrect”) noise model can give a better solution that is closer to the true solution than the intermediate ML-EM solution.

This paper presents a novel concept: There is no universal optimal weighting function for Poisson noise. The so-called “correct” weighting function (*α* = 1) is sub-optimal in almost all cases. The optimal weighting (i.e., the parameter *α*) as well as the iteration number is data count dependent. Algorithm (2) is better than Algorithm (1). We cannot even say that the proposed Algorithm (2) is able to achieve the “ultimate” optimal solution that has the “ultimate” smallest MSE for the given data set, because there may be other noise models or algorithms that may outperform Algorithm (2).

## Figures and Tables

**Fig. 2:**
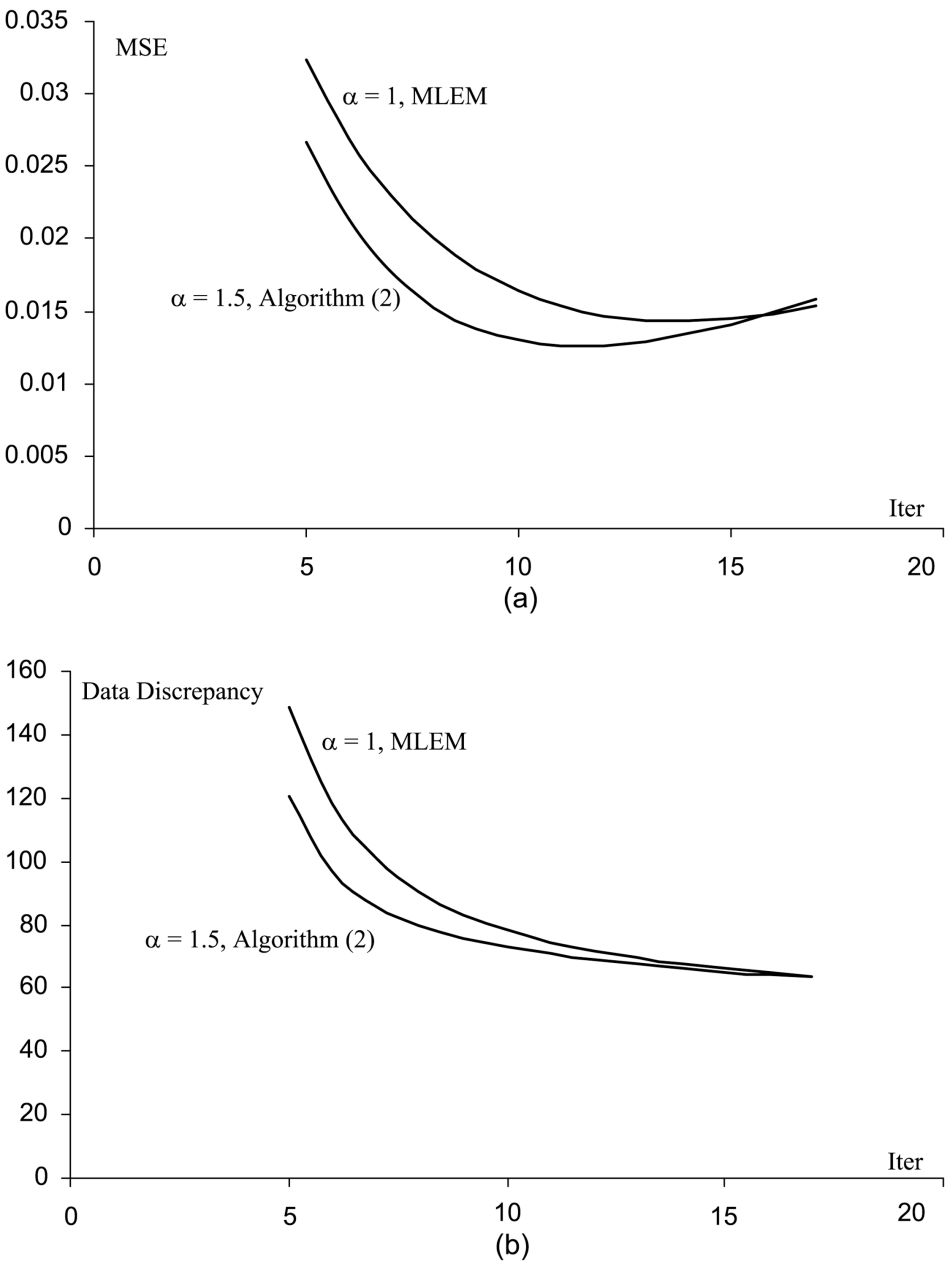
(A) MSE vs. iteration number curves for the MLEM and the Algorithm (2), when the phantom scaling factor is 1 (B) Data fidelity error vs. iteration number curves for the MLEM and the Algorithm (2), when the phantom scaling factor is 1.

**Fig. 3:**
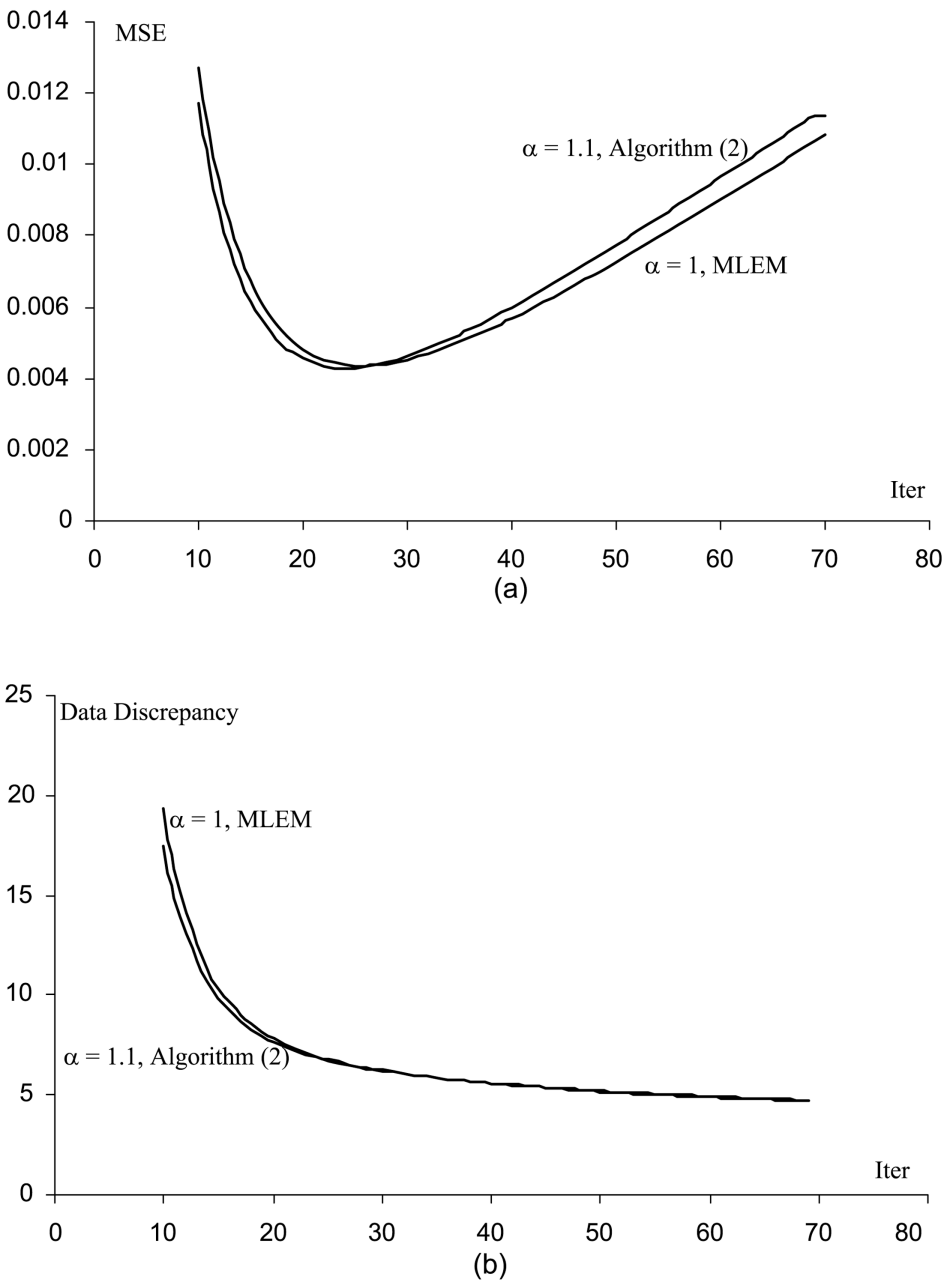
(A) MSE vs. iteration number curves for the MLEM and the Algorithm (2), when the phantom scaling factor is 10 (B) Data fidelity error vs. iteration number curves for the MLEM and the Algorithm (2), when the phantom scaling factor is 10.

**Fig. 4:**
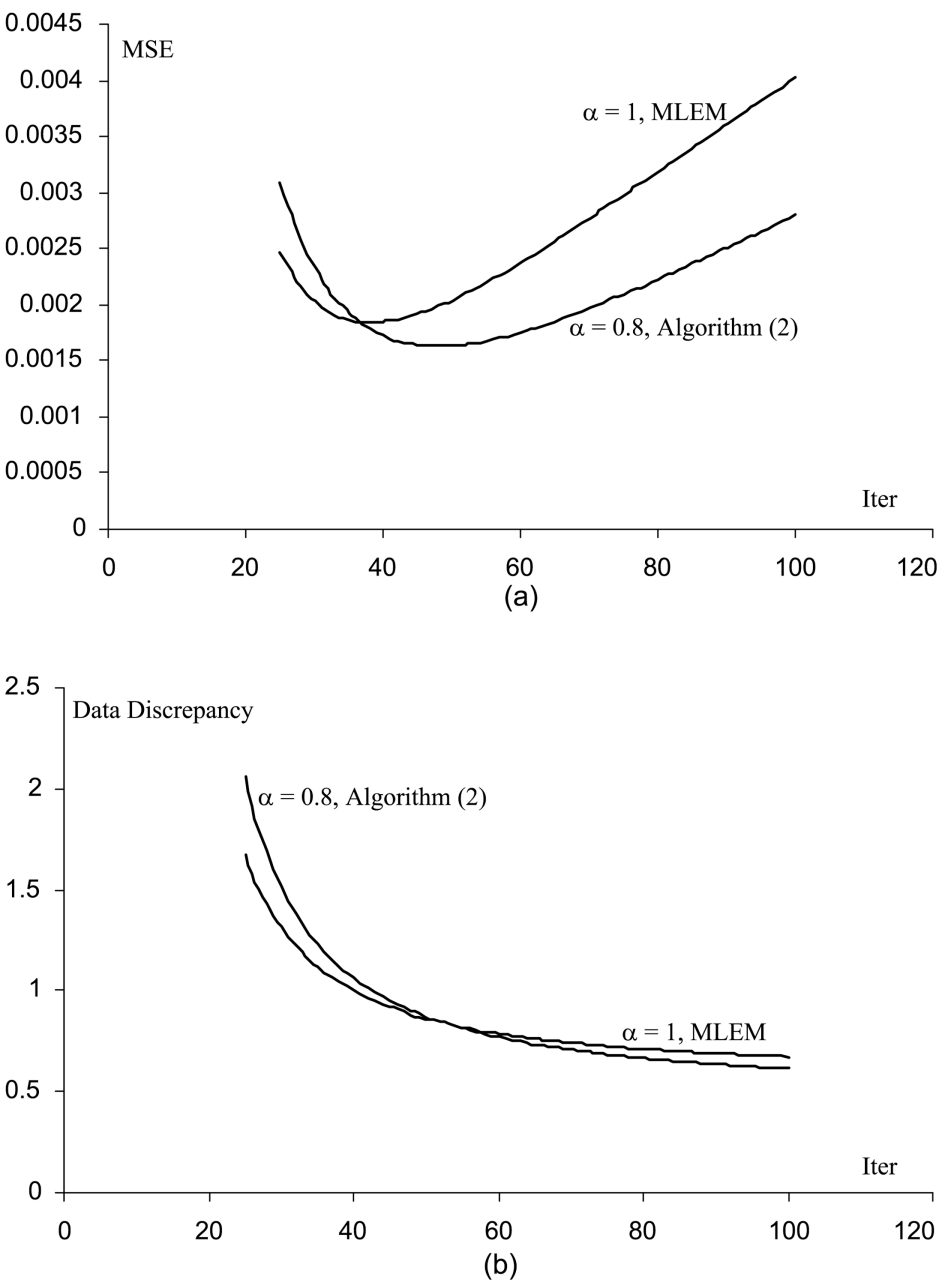
(A) MSE vs. iteration number curves for the MLEM and the Algorithm (2), when the phantom scaling factor is 100 (B) Data fidelity error vs. iteration number curves for the MLEM and the Algorithm (2), when the phantom scaling factor is 100.

**Fig. 5:**
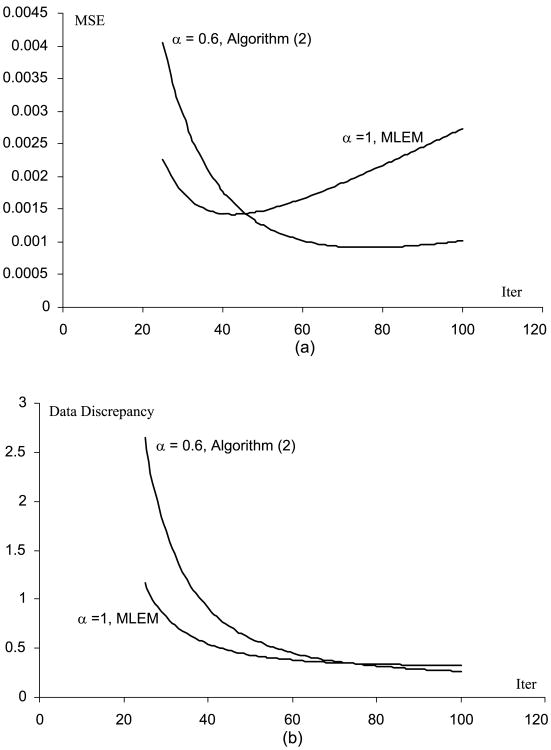
(A) MSE vs. iteration number curves for the MLEM and the Algorithm (2), when the phantom scaling factor is 1000 (B) Data fidelity error vs. iteration number curves for the MLEM and the Algorithm (2), when the phantom scaling factor is 1000.

**Table I:** Best Results for Noise Level 1 where the Data Scaling Factor is 0.1

Algorithm	α	Iter. #	MSE	STDEV	Image	Central horizontal profile

ML-EM(l)	1.0	6	4.042	0.185367	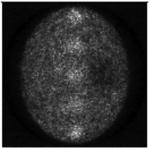	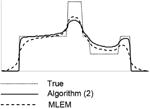
		6	3.962	0.195729		
		6	3.810	0.179073		
		6	3.971	0.180108		
		6	3.820	0.181281		

Algorithm (2)	3.0	4	3.177	0.138820	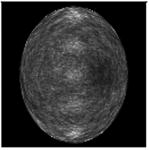	
		4	3.182	0.147451		
		4	3.125	0.136677		
		4	3.196	0.136401		
		4	3.072	0.139339	

**Table II:** Best results for noise level 2 where the data scaling factor is 1

Algorithm	α	Iter. #	MSE	STDEV	Image	Central horizontal profile

ML-EM (1)	1.0	15	1.404	0.115128	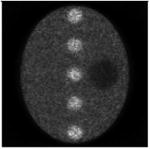	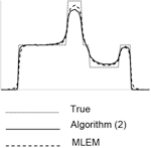
		15	1.385	0.112089		
		15	1.409	0.115123		
		15	1.386	0.115394		
		15	1.431	0.115096		

Algorithm (2)	1.5	12	1.240	0.0947677	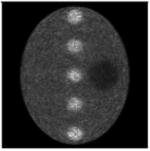	
		12	1.215	0.0930033		
		12	1.233	0.0947398		
		12	1.207	0.0956709		
		12	1.264	0.0948522	

**Table III:** Best Results for Noise Level 3 where the Data Scaling Factor is 10

Algorithm	α	Iter. #	MSE	STDEV	Image	Central horizontal profile

ML-EM (1)	1.0	27	0.4333	0.0615982	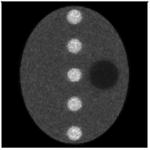	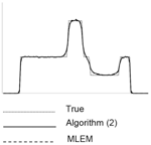
		27	0.4334	0.0587325		
		27	0.4400	0.0605738		
		27	0.4230	0.0596547		
		27	0.4349	0.0610045		

Algorithm (2)	1.1	25	0.4288	0.0579071	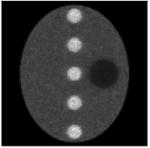	
		25	0.4270	0.0552808		
		25	0.4329	0.0569254		
		25	0.4173	0.0561656		
		25	0.4289	0.0573574	

**Table IV:** Best Results for Noise Level 4 where the Data Scaling Factor is 100

Algorithm	α	Iter. #	MSE	STDEV	Image	Central horizontal profile

ML-EM (1)	1.0	39	0.1815	0.0294572	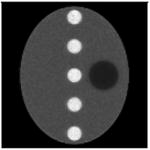	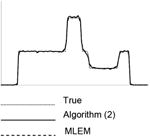
		39	0.1831	0.0284199		
		39	0.1814	0.0297289		
		39	0.1839	0.0278713		
		39	0.1842	0.0294619		

Algorithm (2)	0.8	49	0.1609	0.0343621	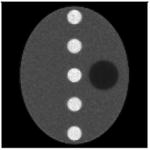	
		49	0.1615	0.0330295		
		49	0.1606	0.0345503		
		49	0.1626	0.0324748		
		49	0.1631	0.0341709		
		

**Table V:** Best Results for Noise Level 5 where the Data Scaling Factor is 1000

Algorithm	α	Iter. #	MSE	STDEV	Image	Central horizontal profile

ML-EM (1)	1.0	44	0.14329	0.0168215	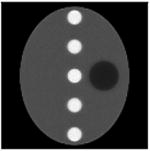	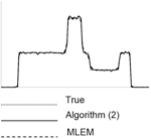
		44	0.14181	0.0164755		
		44	0.14233	0.0166974		
		44	0.14234	0.0196451		
		44	0.14201	0.0166477		

Algorithm (2)	0.6	77	0.09163	0.0220013	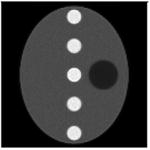	
		78	0.09023	0.0216479		
		77	0.09107	0.0217607		
		78	0.09134	0.0222530		
		77	0.09140	0.0216877	
